# The Digital Abutment Check: An Improvement of the Fully Digital Workflow

**DOI:** 10.1155/2020/8831862

**Published:** 2020-10-24

**Authors:** Sebastian Hinz, Daniel Ellmann, Christian Wegner, Wolfgang Bömicke, Tobias Bensel

**Affiliations:** ^1^Department of Prosthodontics, Faculty of Medicine, Martin Luther University Halle-Wittenberg, Magdeburger Straße 16, 06112 Halle (Saale), Germany; ^2^R+K CAD/CAM Technologie GmbH & Co.KG, Ruwerstieg 43, 12681 Berlin, Germany; ^3^Department of Oral, Dental and Maxillofacial Diseases, Heidelberg University Hospital, Im Neuenheimer Feld 400, Heidelberg 69120, Germany

## Abstract

By using modern digitalization techniques, an existing denture can be digitized and aid the provision of a new implant-supported denture according to a fully digital workflow. This includes fully navigated implant surgery and results in an immediately provided prosthetic restoration. However, even with the current digital workflow, it is challenging to achieve a definitive prosthetic restoration in a single treatment session. In order to achieve a definitive denture in as few treatment sessions as possible, we have implemented the digital abutment test. This test modified the existing data set and determined the final restoration. In the present case, the preexisting maxillary removable complete denture was converted into a fixed immediate restoration using the fully digital workflow. The workflow is divided into two treatment phases, each with three treatment sessions, where part of the second phase involves an innovative digital abutment check. The illustrated case shows an effective use of current digital possibilities. Special attention was also paid to a minimally invasive course of therapy.

## 1. Introduction

Once the hard and soft tissues of the oral cavity have been digitally recorded, e.g., by intraoral scanners and modern 3D radiograph technology, these data can serve as the basis for further digital process steps [1]. Before implant surgery, the final position of the implants can be determined virtually using planning software and 3D radiograph images. The position of the inserted implants is of crucial importance for the final design of the anchored prosthesis and the long-term survival of the dentures [2–4]. In this context, template-guided implant surgery allows for the accurate placement of dental implants [2, 3]. A precise surgical template can be fabricated by merging the intraoral-scan data with the 3D radiographic data [5–7]. For the surgeon, the use of a surgical template results in a predictable and safe treatment procedure. Patients additionally benefit from a shorter treatment time, fewer postoperative restrictions, and an overall increase in comfort [8]. As a consequence, template-guided implant placement is becoming increasingly popular. [8–11].

The implants were placed according to the COMFOUR® system (CAMLOG Vertriebs GmbH, Wimsheim, Germany). Four implants are used to immediately rehabilitate the edentulous jaw with a fixed interim restoration. The advantages of the system lie in the maximum expansion of the support polygon through a precisely planned implant position, but especially implant angulation; the distal implants are usually inserted at an angle between 15 and 30 degrees [3, 4, 12–14]. This allows the prosthetic support field to be expanded posteriorly without compromising relevant anatomical structures and without having to perform extensive bone augmentation measures.

The template used in the present report provided a precise implementation of the digitally determined implant position [5, 9–11, 15, 16]. The navigated procedure offered further advantages by eliminating the need for intraoperative flap formation, thus reducing surgical trauma and reducing the postoperative need for analgesics [8, 17].

However, the main challenge for the prosthetic treatment team (dentist, dental technician) in the implementation of a fully digital All-on-X workflow is to transfer the final implant position to the definitive restoration. The deviations between the virtually planned and the real implant position must be balanced out even with guided implantation [18–25].

Digital impressions represent a reliable impression method for the fabrication of implant-supported full-arch frameworks [26]. Usually, scan-bodies are screwed onto the implants for digital impression making. These can negatively affect the precision of the digital impression [27–29]. However, transmission errors due to the use of scan-bodies must be taken into account. A transfer without the use of scan-bodies could increase the precision of the prosthetic restoration.

In this present case report, we successfully demonstrate the realization of the final prosthodontic restoration while avoiding inaccuracies caused by screwed-in scan-bodies. This was accomplished by the implementation of the digital abutment check without the use of scan-bodies.

## 2. Initial Situation

The 55-year-old patient first came to the Department of Prosthodontics (Faculty of Medicine, Martin Luther University Halle-Wittenberg) during an outpatient consultation. The patient reported smoking about 10 cigarettes a day but otherwise had an uneventful medical history. The maxilla was edentulous, and the remaining teeth in the mandible were stable from a periodontological and endotontological point of view. The existing prosthetic and conservative restorations were found to be sufficient and restored a continuous dental arch, which extended from the second left to the second right premolar.

According to the patient, the last maxillary teeth had been extracted about one year prior to the initial consultation. Since then, he has been wearing a conventionally made removable complete denture. The patient stated that the stability of the prosthesis was not sufficient to eat properly. He also complained about the extensive coverage of the palate. In the course of weighing up the different therapy options, the patient decided on an implant-supported, fixed full-arch prosthesis without palatal coverage. The mandible shortened dental arch was to be maintained in agreement with the patient.

## 3. Therapy Considerations

The aim of the decided upon therapy was to achieve stable, palate-free care, involving little surgical effort and with as few treatment sessions as possible. We decided to use a gentle, minimally invasive procedure without any additional augmentations. The final planning included the insertion of four implants in the upper jaw and a fixed, provisional immediate restoration, which should be transferred to a definitive fixed partial denture (FPD) after the healing period of six months. The treatment was divided into two independent phases. The first treatment phase involves diagnostics and therapy planning, as well as surgical intervention and the immediate provision of an interim restoration. The second treatment phase consists of the transfer of the interim FPD to the definitive FPD after the successful healing period.

We decided to use the COMFOUR® system (Camlog, Wimsheim, Germany) to maximise patient comfort during the treatment. The advantages of the COMFOUR® system are the possibility of immediate loading with appropriate primary stability of the implants (>35 Ncm) and the targeted avoidance of augmentation by angulation of the posterior implants.

## 4. The 1^st^ Treatment Phase (Figure 1)

### 4.1. Pretreatment

As the first treatment step, the mandible and the maxillomandibular relationship were digitally recorded intraorally (Trios 3 intraoral scanner, 3Shape A/S, Copenhagen, Denmark). The correct fit of the maxillary removable complete denture was checked in advance using low viscosity polyvinyl siloxane (GC Fit Checker® Advanced, GC EUROPE N.V., Leuven, Belgium). The correct maxillomandibular relationship was checked clinically based on the resting position of the mandible. If the measured parameters are inadequate, dentures would have to be adjusted in advance. Alternatively, if the patient does not wear any removable dentures or the maxillomandibular relationship has to be changed, the maxillomandibular relationship can be adjusted with digital measurement systems (JMAnalyser+, zebris Medical GmbH, Isny, Germany), due to the fact that these systems offer a digital interface for Computer-Aided Design/Computer-Aided Manufacturing (CAD/CAM).

### 4.2. Preparatory Work

**Figure 2:**
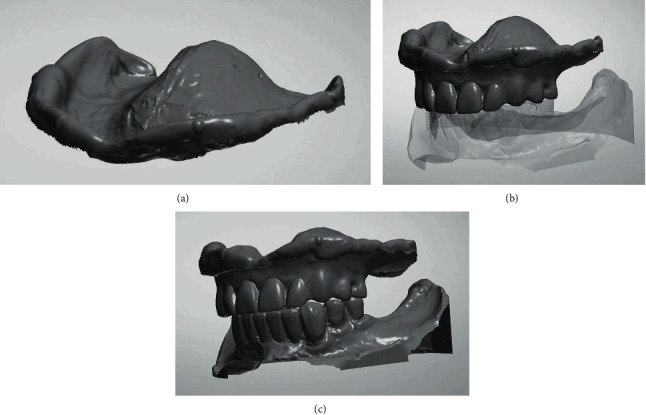
(a) Base area scan of the upper full denture. (b) Scan of the entire upper full denture. (c) Matched scans of the upper full denture and lower jaw.

**Figure 3:**
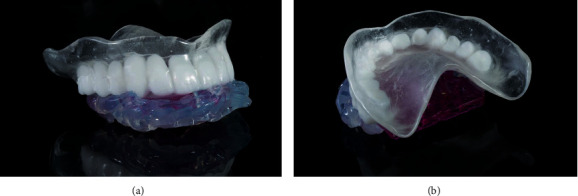
(a) Frontal view of the radiographic template. (b) View on the base area of the radiographic template.

In the present case, the existing maxillary removable complete denture was scanned in the dental laboratory (Rüberling & Klar Dental Laboratory, Halle (Saale), Germany) with a laboratory scanner (E4 lab scanner, 3Shape A/S, Copenhagen, Denmark) (Figure 2). It was then used as a template for the radiographic and the surgical template and the provisional FPD. The Standard Triangulation/Tesselation Language (STL) data records of the mandible and the maxillomandibular relationship record were matched with the data of the existing complete denture (exocad DentalCAD, R+K CAD/CAM Technologie GmbH & Co.KG, Berlin, Germany). The soft tissue situation of the edentulous maxilla was picked up using the base area of the complete denture. This procedure resulted in the manufacturing of the radiographic template. The base of the radiographic template was milled from clear polymethylmethacrylate (PMMA; Organic PMMA clear, Organical Dental Implant, R+K CAD/CAM Technologie GmbH & Co.KG, Berlin, Germany), while the dental arch was milled from barium sulphate containing acrylic resin (Organic RO, Organical Dental Implant, R+K CAD/CAM Technologie GmbH & Co.KG, Berlin, Germany) (Organical® Desktop 8S COMPACT, R+K CAD/CAM Technologie GmbH & Co.KG, Berlin, Germany). Finally, both parts of the radiographic template were connected with an autopolymerizing resin (FuturaGen, Schütz Dental, Rosbach, Germany) (Figure 3).

### 4.3. 3D Radiology

Cone Beam Volumetric Tomography (CBCT) of the upper jaw was performed using the radiographic template. The patient's occlusion was blocked using a silicone occlusion key supplied by the dental laboratory (Transpasil, KANIEDENTA GmbH & Co.KG, Herford, Germany). In this procedure, a Digital Imaging and Communications in Medicine (DICOM®) data record of the upper jaw (Veraview X800, J. MORITA EUROPE GMBH, Dietzenbach, Germany) was created.

### 4.4. Surgical Implant Planning

**Figure 4:**
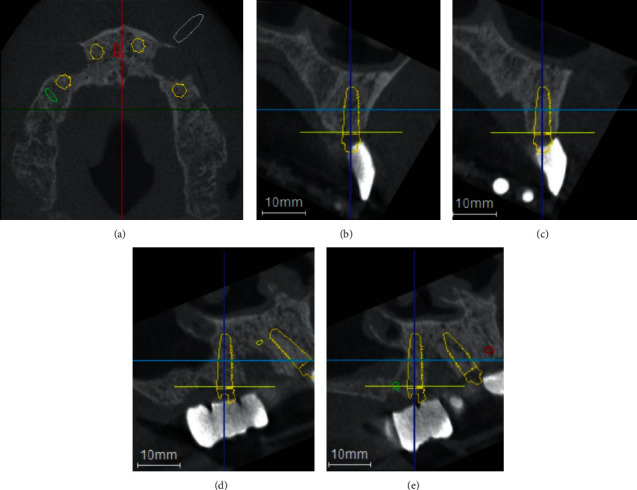
(a) Axial view of all planned implants. (b) Transversal view of planned implant 12. (c) Transversal view of planned implant 22. (d) Transversal view of planned implant 25. (e) Transversal view of planned implant 15.

**Figure 5:**
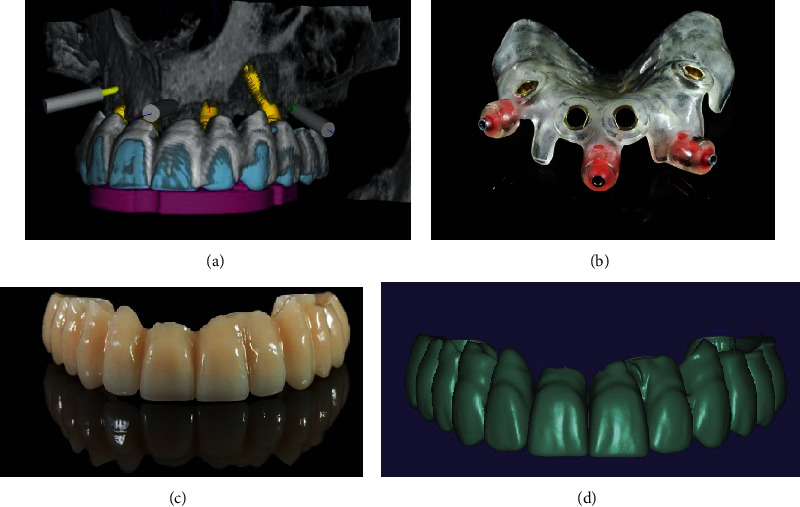
(a) Planning the surgical template. (b) Finished printed surgical template. (c) Design of the provisional FPD. (d) Finished provisional FPD milled from PMMA.

3D planning software (Organical® Dental Implant, R+K CAD/CAM Technologie GmbH & Co.KG, Berlin, Germany) was used for surgical implant planning. In this process, the DICOM® data record and the STL data record were matched. Despite the fact that bony conditions in the anterior maxilla area were reduced, the conditions were sufficient for the insertion of two implants with the size of 3.8 × 11 mm (Guide Camlog®SL Promote plus, CAMLOG Vertriebs GmbH) at the position of the central incisors. In the posterior, the insertion of two implants of size 3.8 × 13 mm was planned in the position of the second premolars (Camlog®SL Promote plus, CAMLOG Vertriebs GmbH). The posterior implants were planned with an insertion angle of 30 degrees. In addition, 3 anchor pins measuring 1.5 × 11 mm at positions 013, 011/021, and 023 were planned for the fixation of the surgical template (Guided Anchor Pin, Nobel Biocare AG, Kloten, Switzerland) (Figure 4). Following the complete surgical implant planning, a corresponding surgical template was printed (VeriGuide™ OS Clear, R+K CAD/CAM Technologie GmbH & Co.KG, Berlin, Germany) (Organical® 3D Print X10 Dentalprinter, R+K CAD/CAM Technologie GmbH & Co.KG, Berlin, Germany) and an interim FPD made of PMMA (Organic PMMA eco A3, Organical Dental Implant, R+K CAD/CAM Technologie GmbH & Co.KG, Berlin, Germany) was milled (Organical® Desktop 8S COMPACT, R+K CAD/CAM Technologie GmbH & Co.KG) (Figure 5).

### 4.5. Implant Surgery

**Figure 6:**
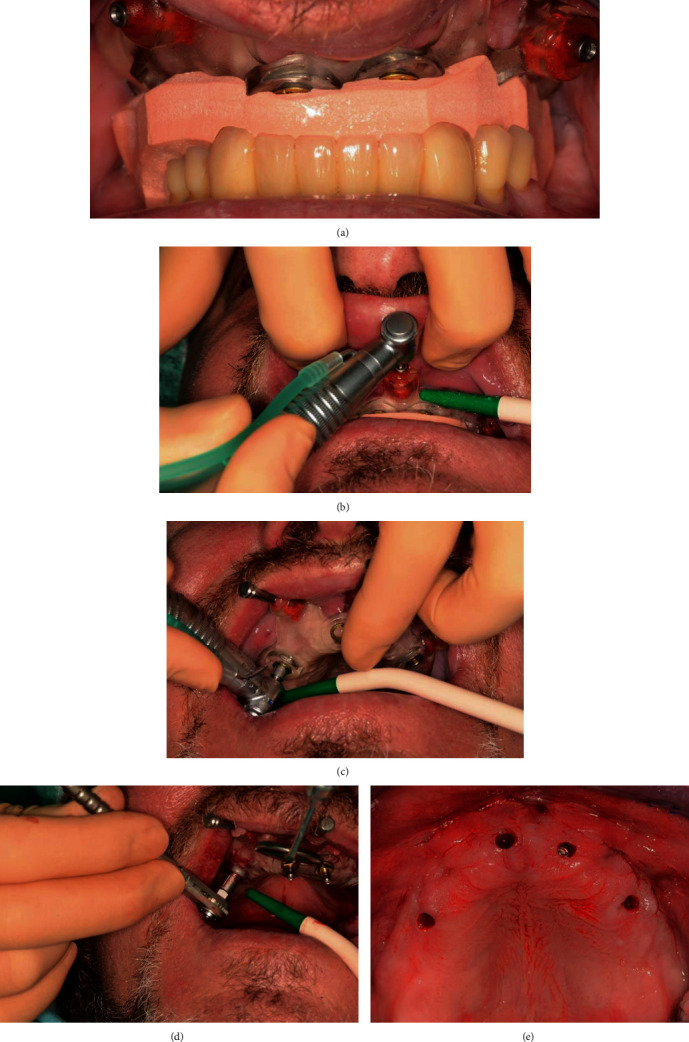
(a) Surgical template with occlusion key. (b) Drilling the holes for the anchor pins. (c) Drilling the implant bearings. (d) Inserting the implants. (e) Upper jaw after implant insertion.

Using a silicone occlusion key (SHERADUETT-SOFT, SHERA Werkstoff-Technologie GmbH & Co.KG, Lemförde, Germany), the surgical template was placed in the patient and fixed in its definite position with anchor pins (Guided Anchor Pin, Nobel Biocare AG). For a flapless surgery, the mucous membrane was punched through the drill sleeves and removed. Afterwards, the implant bearings were reprocessed using 6-13 mm drill bits. For the correct transmission of the planned three-dimensional implant position, the implants (Guide Camlog®SL Promote plus, CAMLOG Vertriebs GmbH) were inserted using the torque wrench up to the marking of the rotation indicator on the drill sleeves. The bone quality corresponded to D2 and the implants performed primary stability (>35 Ncm). Immediate loading was therefore possible (Figure 6).

**Figure 7:**
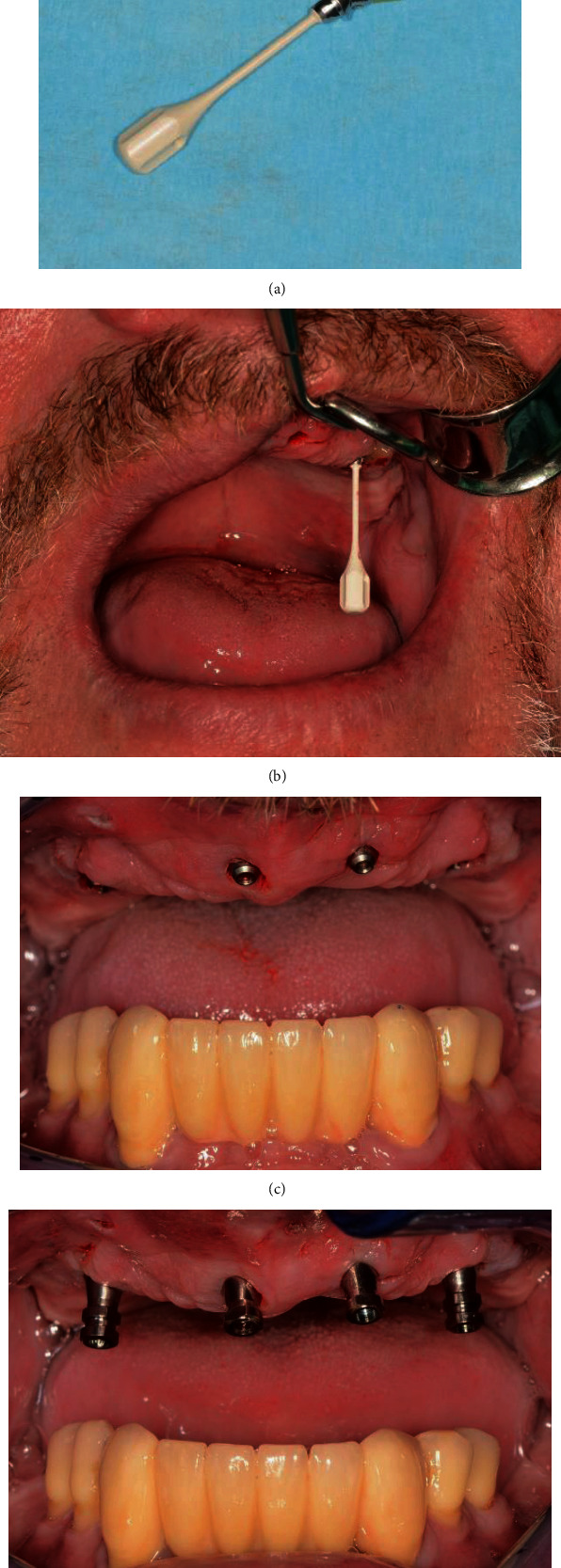
(a) Bar abutment with handle. (b) Inserted bar abutment. (c) Frontal view with inserted bar abutments. (d) Screwed-on adhesive bases.

Abutments compensating for the implant angulation were connected to the implants (bar abutments, CAMLOG Vertriebs GmbH). A flexible handle (COMFOUR®, CAMLOG Vertriebs GmbH) was used to screw in the posterior implants (Figure 7).

### 4.6. Immediate Restoration

Titanium adhesive bases (titanium adhesive base for bar abutment, passive fit, Camlog, Wimsheim, Germany) were screwed onto the bar abutments. This resulted in an intraoral and tension-free bonding of the provisional FPD. The static and dynamic occlusion was checked and adjusted. The provisional FPD was then drained and cleaned using alcohol. Afterwards, the provisional FPD was bonded to the titanium adhesive bases (titanium adhesive base for bar abutment, passive fit, camlog) using autopolymerizing prosthesis repair resin (Qu resin, bredent GmbH & Co.KG, Senden, Germany).

**Figure 8:**
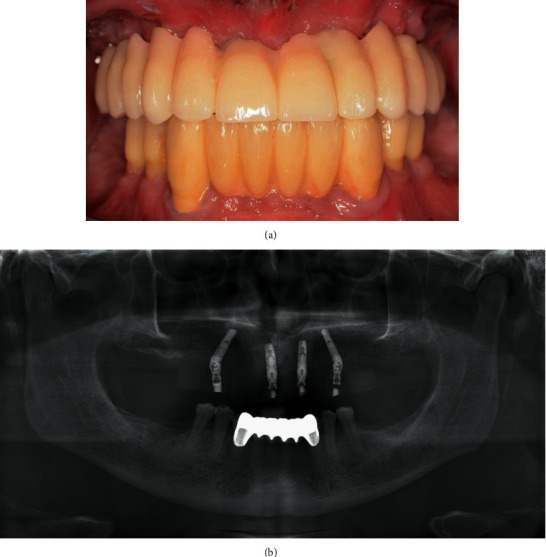
(a) Frontal view of the final inserted provisional FPD. (b) Orthopantomogram to control the implant position.

The basal surface of the interim FPD was elaborated and polished, and then, the interim FPD was tightened to the implants at 15 Ncm and the occlusion finally checked. The screw channels were closed with foam pellets and a gypsum-based sealing material (Cavit™, 3M Deutschland GmbH, Seefeld, Germany), and a postoperative orthopantomogram was then performed (Figure 8).

## 5. The 2^nd^ Treatment Phase (Figure 9)

### 5.1. Abutment Scan

After a six-month implant healing period, the interim FPD had to be replaced by a definitive screw-retained FPD. For this purpose, the occlusion of the existing interim situation and the maxillomandibular relationship were reevaluated.

A digital maxillomandibular relation record was made with the interim restoration in place using an intraoral scanner (Trios 3 intraoral scanner, 3Shape A/S). Then, the interim FPD was unscrewed in order to scan the bar abutments screwed onto the implants and the adjacent soft tissues. After the scan was completed, the provisional FPD was screwed back on.

### 5.2. Abutment Check

**Figure 10:**
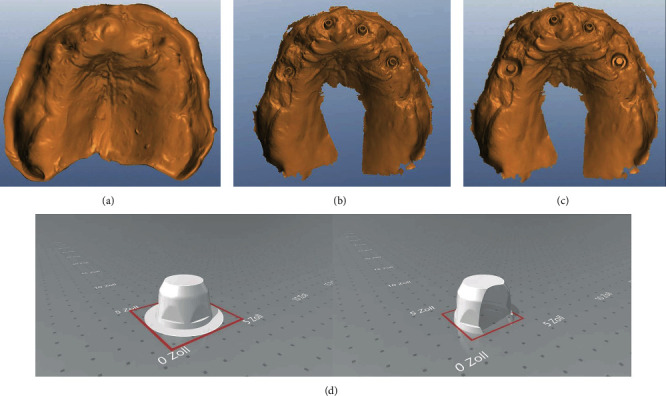
(a) Axial view of the modified upper jaw base initial scan. (b) Axial view of the scanned bar abutments. (c) Axial view of the matched scans. (d) For digital abutment check, the shape of the bar abutments stored in CAD software.

The STL scan data were sent to the dental laboratory for further processing. In the dental laboratory, the existing planning data record is matched with the new maxillomandibular relation record scan and the abutment and soft tissue scan. The incisive papilla, palatine raphe, and palatine rugae served as points of reference for matching the scans. As a result, changes in the jaw relation and soft tissue, as well as minimal positional deviations of the abutments, can be transferred to the definitive FPD (Figure 10).

The cobalt-chromium alloy (CoCrMo) FPD framework (Organic CoCr, Organical Dental Implant, R+K CAD/CAM Technologie GmbH & Co.KG, Berlin, Germany) was designed in accordance with the generated data set (exocad DentalCAD, R+K CAD/CAM Technologie GmbH & Co.KG) and subsequently milled (Organical® 5x dental milling machine, R+K CAD/CAM Technologie GmbH & Co.KG, Berlin, Germany).

### 5.3. Framework Try-In

**Figure 11:**
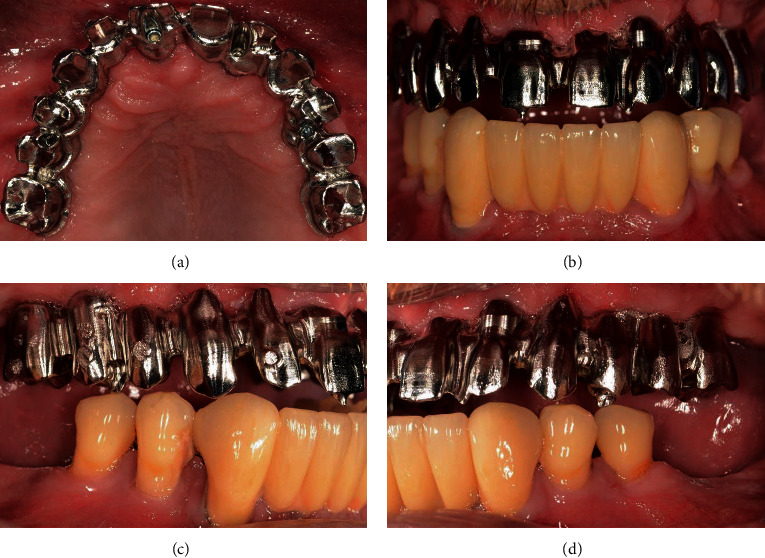
(a) Axial view of the CoCrMo framework. (b) Frontal view of the CoCrMo framework. (c) Right view of the CoCrMo framework. (d) Left view of the CoCrMo framework.

The interim FPD was unscrewed in order to try it in the definitive FPD framework and to check the passivity of fit using the Sheffield test [30]. In the present case, the FPD scaffold fitted without the need for any adjustment (Figure 11). The provisional FPD was then screwed back on, and the definitive FPD scaffold was sent to the dental laboratory for final veneering.

### 5.4. Completion and Inclusion of the FPD

The CoCrMo FPD framework was veneered individually in the laboratory using composite resin material (SR Chromasit, Ivoclar Vivadent, Schaan, Liechtenstein).

**Figure 12:**
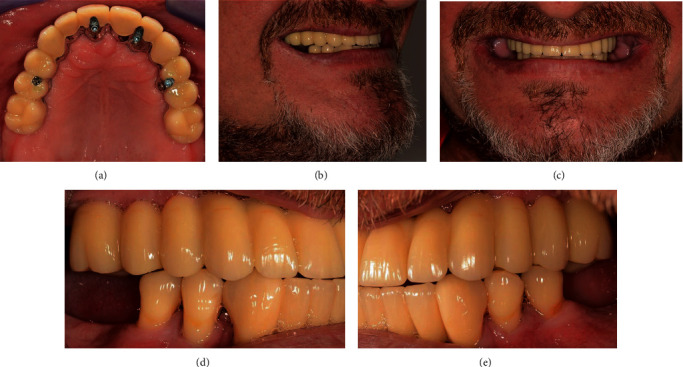
(a) Axial view of the definitive FDP. (b) Right view of the patient smile. (c) Frontal view of the patient smile. (d) Right view of the definitive FDP. (e) Left view of the definitive FPD.

**Figure 13:**
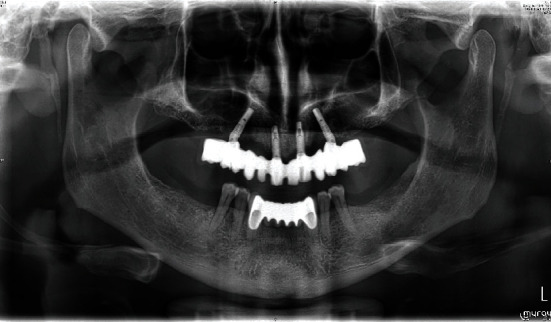
Orthopantomogram to control the definitive FDP.

In the final treatment session, the provisional FPD was removed and the definitive and veneered FPD was screwed on with 15 Ncm. The fit of the FPD was optimal; the occlusion was checked and optimized with minimal grinding measures. Finally, the screw channels were covered with foam pellets and composite resin (CRB-Bonding, SHOFU INC., Kyoto, Japan; Tetric EvoFlow, Ivoclar Vivadent, Schaan, Liechtenstein) (Figure 12). The final orthopantomogram was performed (Figure 13).

## 6. Conclusion

The present case report demonstrates the effective use of the available modern digital manufacturing processes in dentistry. The treatment procedure integrated consequent digital backward planning, fully navigated implantation, and completely digital dental prosthesis production. The procedure described here is in contrast to most of the other All-on-X concepts, which do not involve purely digital processes [6, 7, 12–14].

As conventional impressions could be avoided completely in this workflow, the number of individual treatment sessions (session for maxillomandibular relationship record and try-in) and individual session time could be significantly shortened. For example, the entire surgical procedure up to the installation of the provisional FPD could be carried out by an experienced practitioner in about 75 minutes. The relatively short duration of treatment in combination with a minimally invasive procedure lowers the risk of postoperative complaints such as swelling and pain [8, 17].

The key innovation of this case report is the digital abutment check, which is carried out directly using an intraoral scanner without screwed-in scan-bodies. This is possible because the exact geometry of the bar abutments is stored in the CAD software databases (Organical® Dental Implant, R+K CAD/CAM Technologie GmbH & Co.KG). The direct scan of the bar abutments without the use of scan-bodies again offers advantages in terms of digital impression precision. Possible errors due to incorrect positioning of the scan-body on the implants can thus be excluded [27–29]. Ultimately, this in turn influences the exact fit of the final FPD.

In the present case report, an indication-oriented application of both 3D printing methods and CAM milling methods is also demonstrated. Nowadays, surgical templates can be printed with a clinically acceptable fit [31, 32]. If anchor pins have to be used, the 3D printing process is ideal when compared to milling because even with the most modern 5-axis milling machines, the tool angle is limited. With 3D printing, the drilling channels for the anchor pins, which are often at an angle of 90° to the actual machining axis, can be more easily realized. The printing material, like the milling material, offers the possibility of sterilization before insertion in the patient during the surgery. The ability to sterilize any used dental laboratory materials in order to avoid the chain of infection during the surgical treatment is essential and not just since the beginning of the COVID-19 pandemic situation [33].

The construction of the interim FPD is an additional advantage of the performed digital workflow. The interim FPD was deliberately manufactured using PMMA. In contrast to more stable materials like zirconium dioxide, PMMA offers potential material-specific benefits. These benefits result in the ability to check and adapt the maxillomandibular relationship during the provisional restoration phase. This means that the occlusion of the provisional PMMA-FPD could be either easily reduced by grinding or increased by adding self-curing resin material. The higher abrasiveness of the material offers some protection against overloading the implants during the healing process.

Finally, the definitive FPD may be veneered with ceramic instead of composite resin. In contrast, it is also possible to produce a fully anatomical milled FPD, which can be inserted as the definitive dental restoration. This procedure could avoid the previous scaffold try-in. In this case report, one of the objectives of the treatment procedure was that the denture should be easily repairable. Therefore, the veneering material chosen for the definitive FPD was composite resin. However, compared to ceramic veneering material, composite resin enables easier occlusal adaptation to the opposing jaw.

In contrast to a conventional treatment process, not only are sessions for the maxillomandibular relationship and possibly the scaffold try-in avoided, but the process described here also creates an accurate adjustment of the definitive restoration. The treatment concept shown in this present case report combines a safe and time-saving digital workflow with demanding, predictable therapy results. In addition, the surgical intervention was not a major burden for the patient. The enormous gain in quality of life exceeds the manageable treatment effort for the patient and is clearly in focus.

The patient's wish to switch from a removable complete denture to a fixed, palate-free prosthetic restoration could be fulfilled after three sessions following the end of the first treatment phase. In addition, both the original tooth position and aesthetics could be transferred to the provisional and definitive FPD, producing a harmonious appearance that was familiar to the patient.

## Figures and Tables

**Figure 1:**
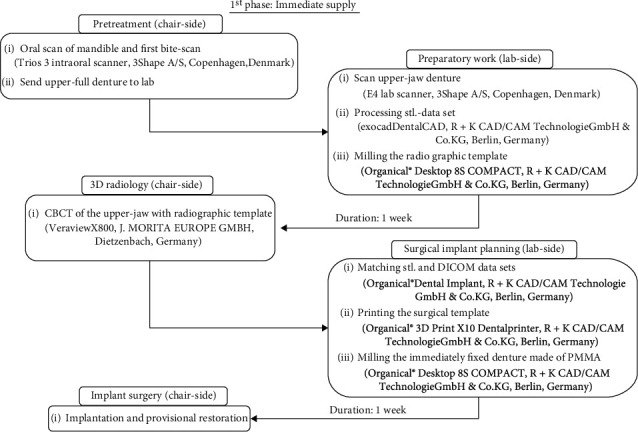
Overview of the 1^st^ treatment phase.

**Figure 9:**
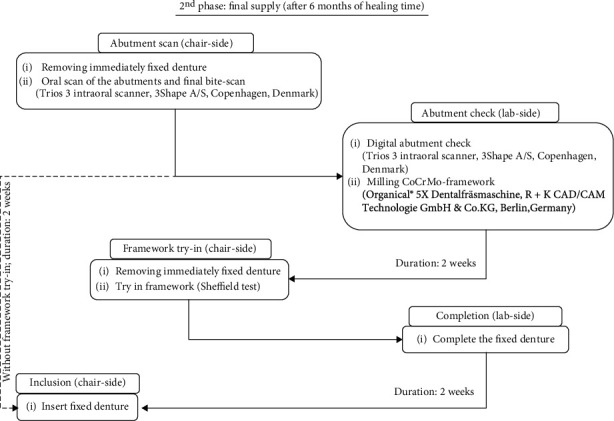
Overview of the 2^nd^ treatment phase.

## Data Availability

The data used to support the findings of this study may be released upon application to the Department of Prosthodontics, Martin-Luther-University Halle-Wittenberg, which can be contacted at Dr. Christian Wegner, Department of Prosthodontics, University Hospital Halle, Magdeburger Straße 16, 06112 Halle (Saale), Germany.
